# Comparison of the Effects of Acute Appendicitis and Chronic Cholecystitis on Intestinal Mucosal Function During Surgery

**DOI:** 10.7759/cureus.30953

**Published:** 2022-11-01

**Authors:** Danyang Wang, Liuxu Yao, Rui He, Suqin Huang, Zeyong Huang, Kai Fang, Yuhong Li

**Affiliations:** 1 Department of Anesthesiology, Shulan (Hangzhou) Hospital, Hangzhou, CHN; 2 Department of Anesthesiology, Zhejiang Provincial People's Hospital, Affiliated People's Hospital, Hangzhou Medical College, Hangzhou, CHN; 3 Department of Anesthesiology, Shaoxing People's Hospital, Hangzhou, CHN; 4 Department of Anaesthesiology, Shulan (Hangzhou) Hospital, Hangzhou, CHN

**Keywords:** intestinal permeability, intestinal mucosal function, inflammatory factors, chronic cholecystitis, acute appendicitis

## Abstract

Objective: The objective of this study is to explore the effect of acute appendicitis and chronic cholecystitis on inflammatory factors and intestinal mucosal function during operation.

Methods: This was a prospective cohort study. Forty-four patients including those who underwent elective cholecystectomy (Group A, n = 22) or emergency appendectomy (Group B, n = 22) were recruited. Before anesthesia and after surgery, arterial blood was collected for the measurement of plasma indices associated with inflammation or intestinal permeability.

Results: Both the tumor necrosis factor-alpha (TNF-α) and C-reactive protein (CRP) levels were higher in Group B than in Group A (P < 0.05). The preoperative IL-10 level was higher in Group A than in Group B (P = 0.036), while after surgery, the opposite relationship was observed (P = 0.020). There were no intergroup or intragroup differences for D-Lac. The postoperative lipopolysaccharide (LPS) and human syndecan-1 (Sdc-1) levels were lower than the corresponding preoperative value (P < 0.05) in the two groups. Both the preoperative Sdc-1 and fatty acid binding protein (FABP2) levels in Group A were higher than the corresponding levels in Group B (P < 0.05).

Conclusions: The study suggested that chronic cholecystitis had more severe damage to intestinal mucosal function than acute appendicitis. It is necessary to strengthen the protection of intestinal mucosa during the perioperative period.

## Introduction

Cholecystolithiasis is a common clinical gallbladder disease, which causes acute and chronic cholecystitis and often requires surgical treatment; acute appendicitis is a common surgical abdominal emergency in general surgery, and surgical treatment should be carried out as soon as possible after diagnosis. Laparoscopic cholecystectomy or appendectomy is the main surgical treatment for the two diseases. There were different degrees of infection in patients with two kinds of diseases before the operation. Previous studies have suggested that acute and chronic inflammation can lead to impaired barrier integrity in the intestinal mucosa, which demonstrated the increase of intestinal mucosal permeability [1]. Studies have shown that increased intestinal mucosal permeability is not only associated with intestinal bacterial/toxin migration resulting in inflammation in distant organs but also correlated with disease prognosis [1-3]. Therefore, scholars have proposed that intestinal mucosal permeability is a new target for disease prevention and treatment [4]. Surgical stress, anesthesia, and trauma may lead to increased postoperative intestinal mucosal permeability [5,6]. In the case of intestinal surgery due to a tumor or acute infectious disease, hyperpermeability of the intestinal mucosa most likely occurs [7]. Our recent study used volume kinetics to demonstrate that after emergency surgery for acute appendicitis, fluid distributed to the interstitial space from the vascular space at a constant rate (*k_12_*) after the infusion of crystalloid, whereas fluid returned to the vascular space at a different constant rate (*k_21_*). The patients with appendicitis had a* k_21_* that was relatively slower than their *k_12_* [8]. Large animals in shock-like states [9] or patients with transurethral prostatectomy syndrome [10] have similar manifestations, so it is speculated that *k*_*21* _may be an early sign of disease progression. The most reliable explanation is fluid accumulation in the intestinal mucosa. So whether there are differences between the two diseases in intestinal mucosal permeability during operation has not been fully clarified. However, whether there are differences in the effects of the above two diseases on inflammatory factors and intestinal mucosal permeability has not been fully elucidated.

According to previous studies, the following hypotheses were put forward: The level of inflammatory cytokines in acute appendicitis was higher than that in patients with chronic cholecystitis; there were differences in the effects of the two diseases on intestinal mucosal function. In order to verify this assumption, patients with acute appendicitis or chronic cholecystitis were included. Laparoscopic appendectomy or elective cholecystectomy under general anesthesia was performed. The levels of inflammatory factors and cytokines related to intestinal mucosal function were detected before and after the operation. The results provide the basis for different intestinal function protection strategies in the perioperative period of the two diseases.

## Materials and methods

Ethics and patients

The study was reviewed and approved by the Shaoxing People’s Hospital Review Board. The Ethics Committee of Shaoxing People's Hospital approved the experimental protocol (no. 2015013; official in charge: Yu Qian), and the study was registered with the Chinese Clinical Trial Registry (website: http://www.clictr.org.cn, no. CHICTR-15006063). All patients or their guardians were fully informed of the protocol and signed the consent form before participating in the trial. Between March 2015 and April 2017, 44 patients aged 18-60 years with body mass index (BMI) ranging from 18 to 25 and physical status I-II according to the American Society of Anesthesiologist (ASA) who were diagnosed with chronic cholecystitis undergoing elective laparoscopic cholecystectomy (Group A, n = 22) or acute appendicitis undergoing emergency laparoscopic appendectomy (Group B, n = 22) under general anesthesia were included in the study. Patients with septic shock; cardiopulmonary, hepatorenal, or endocrine diseases; severe anemia (Hb < 6.5 g/dL); nervous system diseases; intraoperative blood loss greater than 200 mL; vasopressor-dependent low blood pressure; or surgical procedure changes were excluded. Additionally, patients with preoperative cognitive impairment, obese patients (BMI > 25), and pregnant or lactating women patients were also excluded.

Drugs, kits, and equipment

Ringer's lactate solution (Baxter Healthcare Limited, Shanghai, China; H10983055 approved by the state); injectable propofol (AstraZeneca UK Limited; H20100646 approved by the state); midazolam (Enhua Pharmaceutical Co., Ltd, Jangsu, China); cisatracurium besylate injection (Jiangsu Hengrui Pharmaceutical Co., Ltd., Lianyungang, China; H200620869 approved by the state); sufentanil citrate injection (Yichang Renfu Pharmaceutical Co., Ltd, China; H20054171 approved by the state); sevoflurane (Jiangsu Hengrui Pharmaceutical Co., Ltd, Lianyungang, China; H20040771 approved by the state); human tumor necrosis factor-alpha (TNF-α) and interleukin-10 (IL-10) enzyme-linked immunoassay (ELISA) kits (MultiScience Biotech Co., China); human syndecan-1 (Sdc-1), lipopolysaccharide (LPS), and intestinal fatty acid binding protein (IFABP or FABP2) ELISA kits (Clone Corp., TX, USA); a D-lactic acid ELISA kit (Abebio, USA); C-reactive protein (CRP) (MedicalSystem Biotechnology Co., Ltd., China; 20172400911 approved by the state); a multifunction monitor (Datex Ohmeda, Netherlands); an arterial blood gas analyzer (GEM Premier 3000, Instrumentation Laboratory, Illinois, USA); an anesthesia depth monitor (a-2000TM, Aspect Medical System, USA); and a multiband microplate reader (SpectraMax Plus, Molecular Devices, USA) were used in this study.

Procedure

**Figure 1:**
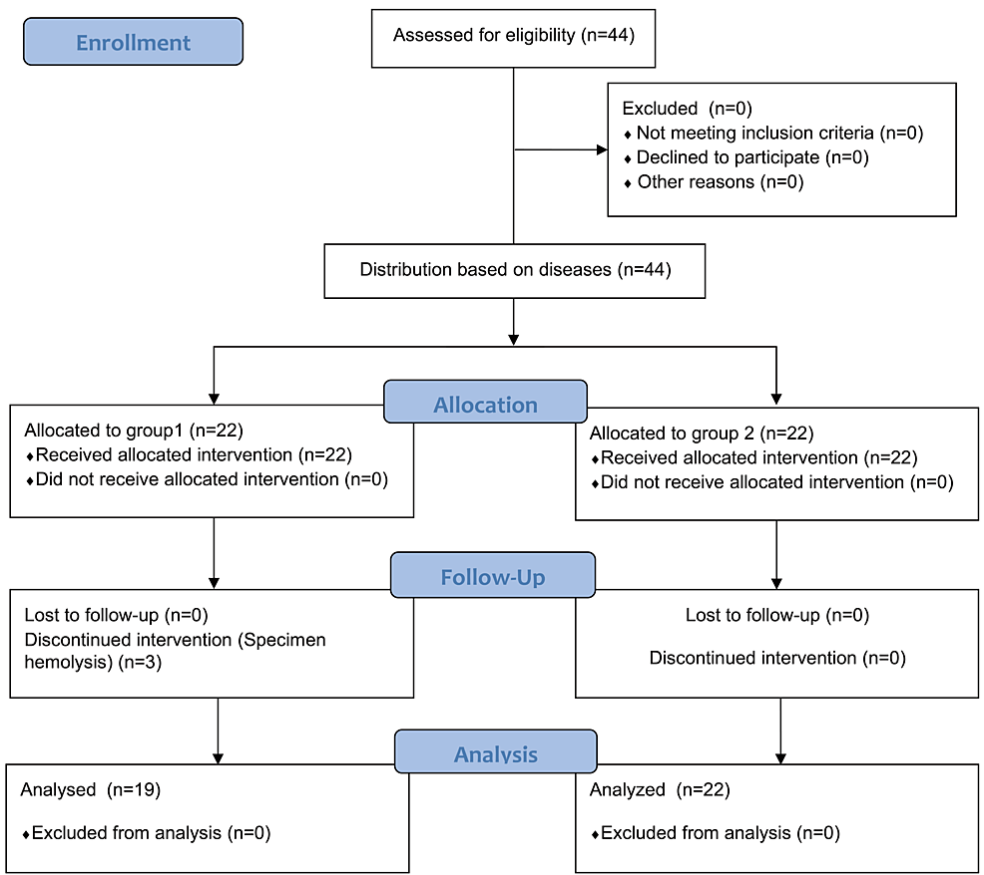
Flow diagram

This study was completed in the operating room and the clinical research center of Shaoxing People’s Hospital. All of the patients fasted for eight hours before surgery without preoperative medication. After entering the operating theater, all of the patients were given oxygen at a rate of 2-4 L/h. A catheter was placed in the left radial artery to monitor blood pressure and collect blood samples for measurements. Anesthesia was induced with propofol (1.5 mg/kg), midazolam (50 μg/kg), cisatracurium (0.15 mg/kg), and sufentanil (5 μg/kg). After tracheal intubation, mechanical ventilation parameters were set as follows: positive end-expiratory pressure of 3 cm H_2_O, and I:E ratio of 1:2. Tidal volume (VT) or breathing frequency is regulated to maintain the end-expiratory carbon dioxide pressure at 36-44 mmHg. Anesthesia was maintained with 1%-2% sevoflurane and 6 mg/kg/h propofol. Additional sufentanil (0.2-0.4 μg/kg) and cisatracurium (0.05 mg/kg) were administered as required. The depth of anesthesia was monitored by using a bispestral index (BIS) sensor applied to the forehead, with a BIS value between 40 and 60. After the induction of anesthesia, every patient was administered Ringer’s lactate solution at a dose of 15 mL/kg. During the experiment, ephedrine (5-10 mg) was administered i.v. when the mean arterial pressure (MAP) fell to less than 65 mmHg, and atropine (0.5 mg) was administered i.v. when the heart rate was less than 50 beats/min. Patients were transferred to the postoperative anesthesia care unit after surgery (Figure 1).

Specimen collection and indicators

Three milliliters of arterial blood were collected both before anesthesia and after surgery and placed in a tube containing ethylenediaminetetraacetic acid (EDTA), centrifuged for 30 mins at 1000 g, and stored at minus 80℃. The plasma concentrations of TNF-α, IL-10, Sdc-1, LPS, FABP2, and D-lactic acid were measured by ELISA according to the kit instructions. The plasma CRP concentration was measured by immunoturbidimetry. Preoperative and one-day postoperative blood routine tests and biochemical results were collected from the hospital information system to evaluate postoperative complications.

Statistical analysis and sample size estimation

Statistical analyses were performed with Statistical Package for the Social Sciences (SPSS) software (version 18) for Windows (SPSS Inc., Chicago, IL) and GraphPad Prism software version 7 (GraphPad Software Inc., USA). Normally distributed numerical variables are presented as the mean ± standard deviation (SD) and were analyzed by student’s t-test, while nonnormally distributed variables are presented as the median (interquartile range) and were analyzed by the Mann-Whitney U test. Incidence data are presented as a number or percentage and were analyzed by the chi-square or Fisher's exact test when appropriate. A P-value less than 0.05 was considered statistically significant.

Sample size estimation was based on an SD that was 1/3 of the mean, according to the trial study. Power was set at 0.85 to detect a 35% difference at P < 0.05 with the use of G*Power software, version 3.0.10. It was then estimated that a minimum of 19 subjects would be necessary. Twenty-two subjects were recruited for each group.

## Results

Demographic data, fluid input, and output

Forty-one patients completed the clinical trial, including 22 patients in Group A who underwent laparoscopic cholecystectomy for cholecystitis and 19 patients in Group B who underwent laparoscopic appendectomy for acute appendicitis (Figure 1). There were no statistically significant differences between the two groups in terms of general patients characteristics, such as age (46 ± 11 yrs vs 47 ± 16 yrs, P = 0.831), body weight (59 ± 13 kg vs 57 ± 9 kg, P = 0.537), height (163 ± 10 cm vs 164 ± 9 cm, P = 0.816), and BMI (22 ± 4 vs 21 ± 3, P = 0.652). The operation time (54 ± 22 min vs 44 ± 17 min, P = 0.110), urine output (1.4 [0.9, 2.2] mL/kg vs 1.4 [0.9, 1.6] mL/kg, P = 0.948), blood loss (20 [20, 25] mL vs 20 [20, 20] mL, P = 0.821), and infusion volume (878 ± 196 mL vs 857 ± 128 mL, P = 0.716) were also comparable between the two groups (P > 0.05).

Plasma inflammatory mediators' concentration before and after the operation

**Figure 2:**
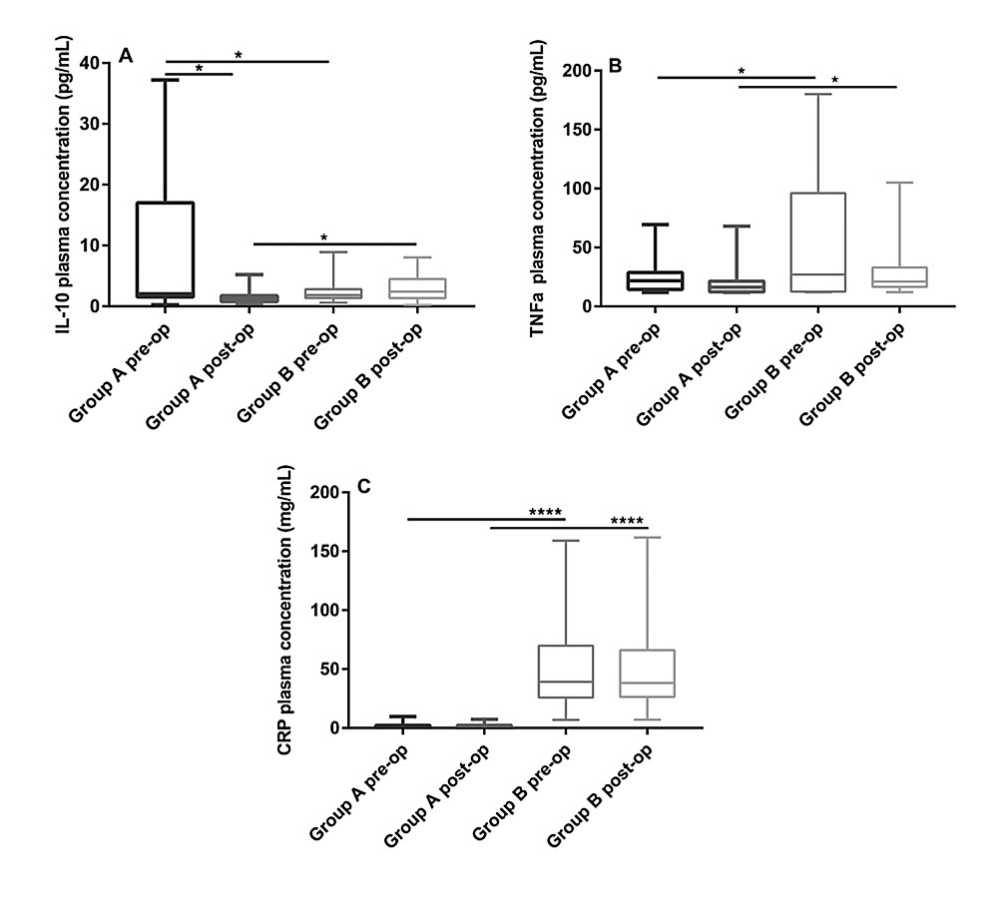
Comparisons of the IL-10 (A), TNF-α (B), and CRP (C) plasma concentrations Data are expressed as the median (interquartile range). *P < 0.05, ****P < 0.0001. TNF-α: Tumor necrosis factor-alpha; CRP: C-reactive protein.

The postoperative IL-10 plasma concentration in Group A was lower than the preoperative concentration (1.25 [0.88, 1.71] pg/mL vs 2.05 [1.57, 9.01] pg/mL, P = 0.015), while in Group B, the preoperative and postoperative IL-10 concentrations were similar (1.86 [1.40, 2.75] pg/mL vs 2.41 [1.62, 3.70] pg/mL, P = 0.579). Comparing the preoperative level between the two groups showed that the IL-10 level in Group B was lower than that in Group A (1.86 [1.40, 2.75] pg/mL vs 2.05 [1.57, 9.01] pg/mL, P = 0.036), and comparing the postoperative levels showed the opposite pattern (2.41 [1.62, 3.70] pg/mL vs 1.25 [0.88, 1.71] pg/mL, P = 0.024) (Figure 2, Panel A).

The postoperative TNF-α plasma concentrations in both groups were lower than the corresponding preoperative levels, but the differences were not statistically significant (P > 0.05). However, the preoperative (27.42 [12.14, 86,35] pg/mL vs 21.81 [14.78, 27.34] pg/mL, P = 0.042) and postoperative (20.99 [19.04, 34.92] pg/mL vs 16.24 [12.48, 21.22] pg/mL, P = 0.011) TNF-α plasma concentration in Group B were higher than those in Group A (Figure 2, Panel B).

The postoperative CRP plasma concentrations were comparable with the preoperative levels in both groups (P > 0.05), while the preoperative (39.26 [27.22, 65.37] mg/mL vs 1.25 [0.47, 2.24] mg/mL, P < 0.0001) and postoperative (28.32 [27.01, 64.40] mg/mL vs 1.14 (0.47, 2.16) mg/mL, P < 0.0001) CRP plasma concentrations in the Group B were significantly higher than those in Group A (Figure 2, Panel C).

Plasma concentrations of intestinal permeability-related indexes

**Figure 3:**
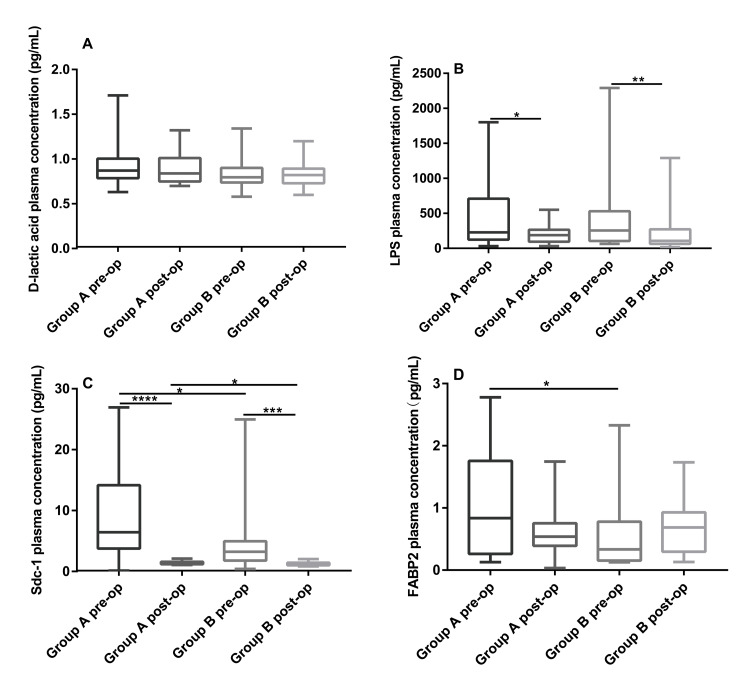
Comparisons of the D-lactic acid (A), LPS (B), Sdc-1 (C), and FABP2 (D) plasma concentrations Data are expressed as the median (interquartile range). *P* *< 0.05; **P* *< 0.01; ***P* *< 0.001; ****P* *< 0.0001. LPS: Lipopolysaccharide; Sdc-1: Human syndecan-1; FABP: Fatty acid binding protein.

There were no statistically significant differences in the D-lactic acid plasma concentrations within the two groups before and after the surgery or between the two groups before and after the surgery (P > 0.05) (Figure 3, Panel A). The postoperative plasma concentrations of LPS in the two groups were decreased significantly compared with the preoperative levels (in Group A: 212.0 [120.56, 576.55] pg/L vs 180.6 [62.79, 272.34] pg/mL, P = 0.014; in Group B: 255.8 [104.60, 531.23] pg/mL vs 106.8 [62.82, 272.33] pg/mL, P = 0.009). However, there were no significant differences in the LPS plasma concentration between the two groups before and after surgery (P > 0.05) (Figure 3, Panel B).

The postoperative plasma Scd-1 concentration in both groups showed downward trends with high statistical significance (in Group A: preoperative 6.71 [3.60, 14.97] ng/mL vs postoperative 1.40 [1.18, 1.59] ng/mL, P < 0.001; in Group B: 3.23 [1.80, 4.97] vs 1.13 [1.01, 1.44], P = 0.0008). Preoperative (6.71 ng/mL vs 3.23 ng/mL, P = 0.020) and postoperative (1.40 [1.18, 1.59] vs 1.13 [1.01, 1.44], P = 0.012) comparisons between the two groups showed that the plasma concentrations of Scd-1 in Group A were significantly higher than those in Group B (Figure 3, Panel C).

The FABP2 plasma concentrations in the two groups showed opposite trends before and after the surgery. Specifically, the preoperative FABP2 level in Group A was higher than the postoperative level (0.80 [0.26, 1.76] ng/mL vs 0.53 [0.39, 0.75] ng/mL, P = 0.111), while the preoperative level in Group B was lower than the postoperative level (0.33 [0.15, 0.78] ng/mL vs 0.69 [0.30, 0.93] ng/mL, P = 0.284); however, there were no significant differences. Comparisons of the preoperative or postoperative FABP2 plasma concentration between the two groups showed that the FABP2 level in Group A was significantly higher than that in Group B (0.80 [0.26, 1.76] ng/mL vs 0.33 [0.15, 0.78], P = 0.042) before surgery, whereas the opposite relationship was observed after surgery, but the difference was not significant (0.53 [0.39, 0.75] ng/mL vs 0.69 [0.30, 0.93] ng/mL, P = 0.611) (Figure 3, Panel D).

## Discussion

The surgical procedures for the two kinds of diseases were similar, and there was no significant difference in the demographic data, operation time, fluid intake, and output between the two groups.

The results showed that the changes in the levels of the proinflammatory factors TNF-α and CRP were consistent. There were decreasing trends in the two groups post-operation compared with pre-operation (not statistically significant). TNF-α is mainly produced by activated lymphocytes, macrophages, and monocytes. The level of TNF-α in vivo is closely related to the degree of infection [11,12]. No matter before or after the operation, the two kinds of proinflammatory factors in patients with acute appendicitis are higher than those in patients with chronic cholecystitis, which indicates that the inflammatory response in acute appendicitis was stronger than that in chronic cholecystitis. The changes in the level of the anti-inflammatory mediator IL-10 showed different trends. The plasma concentration of IL-10 was higher in Group A than in Group B before surgery, while the opposite was true after surgery. It may be because chronic inflammation in the gallbladder constantly stimulates the body to produce a higher concentration of anti-inflammatory mediators. The mechanism responsible for the IL-10 level being higher in Group B than in Group A after surgery remains unclear, and further research is required.

Acute appendicitis accompanied by systemic infection may cause varying degrees of damage to the intestinal mucosal barrier and increase intestinal mucosal permeability [9,10]. In addition, intraoperative mechanical overstretch and direct intestinal injury caused severe intestinal mucosal ischemia, which aggravated the injury of the intestinal mucosa. Even in nonintestinal surgery, the permeability of the intestinal mucosa can also increase. In abdominal, especially intestinal, surgery, there is a direct injury of the intestinal mucosa, which aggravates intestinal mucosal ischemia and intestinal mucosal injury; even if nonintestinal surgery, intestinal mucosal permeability may also increase [2]. Our previous studies [13,14] found that the amount of intravenous infusion of fluid excreted through the kidneys for patients under anesthesia was only 10% of that excreted by conscious volunteers. Thus, the infused fluid stays in the intravascular space, resulting in volume expansion, or it can accumulate in the extravascular space, which is one of the causes of postoperative edema. Studies have confirmed that intraoperative fluid overload that results in weight gain after surgery is associated with postoperative morbidity and mortality [15,16]. Volume kinetic analysis [8,9] showed that in the patients with inflammation, the fluid transport constant rate from the interstitial space to the central volume space (*k_21_*) was nearly zero or even negative. Combined with anesthesia and surgical effects, even restricted infusion can result in fluid accumulation in the interstitial space, resulting in tissue edema. The increase in* k_^21^_* is related not only to the increase in the hydrostatic pressure of the capillaries but also to the permeability of the capillaries. Therefore, a low value of *k*_*21* _might be an early sign of severe disease. A plausible explanation could be that *k_21_* might be an indicator of intestinal mucosal permeability. The specific mechanism remains unclear.

A recent study showed that Aquaporin 3 (Aqp3) is involved in the regulation of pulmonary vascular permeability and that antioxidants can reduce pulmonary permeability and downregulate the expression of Aqp3 in sepsis [17]. Another study found that Aqp3 can maintain the integrity of the intestinal mucosal barrier. After knocking out Aqp3, the integrity of the intestinal mucosal barrier is damaged, and intestinal mucosal permeability increases [18]. Intestinal mucosal permeability is related to disease prognosis [1-3] and might be one of the targets of disease prevention and treatment [4]. Therefore, the indicators of intestinal mucosal permeability have received increased attention. The ideal monitoring indicators depend not only on their specificity for the intestinal tissue but also on their location, content, release mechanism, stability, and serum clearance. Recent clinical studies have shown that FABP2 appears early in the disease and exhibits strong specificity compared with traditional serological indicators and is considered to be the most ideal serological marker for the diagnosis of intestinal ischemia [19,20]. FABP2 is a unique protein, which is approximately 1%-2% of the total cytoplasmic protein, and it exists in the cytoplasm of intestinal villous epithelial cells.

In the early stage of intestinal ischemia, the integrity of intestinal epithelial cells is compromised, which results in the release of FABP2 into the blood. The concentration of FABP2 is relatively stable for 24 hours at room temperature, facilitating detection. It is higher in the urine than in the blood, thus the intestinal mucosal permeability of patients can be monitored by detecting the concentration of FABP2 in the urine. Normally, FABP2 can be cleared quickly (*T_1/2 _*= 11 min); thus, an increased serum FABP2 concentration reflects persistent intestinal mucosal injury. D-lactic acid in the human body is mainly produced via fermentation by gastrointestinal bacteria. As mammals cannot metabolize D-lactic acid, the factors causing the increase in intestinal permeability will lead to an increase in the plasma D-lactic acid level. Therefore, an increase in the D-lactic acid concentration reflects an increase in bacteria or their metabolites entering the circulation, which is a marker of intestinal bacteria translocation, which indirectly reflects the functional status of the mechanical intestinal barrier [21] and can become a biochemical marker of acute intestinal ischemia due to its high sensitivity and specificity [22].

The present study showed that there were no significant intragroup or intergroup changes in the plasma D-lactic acid, but the plasma concentration of FABP2 exhibited different trends. Before surgery, the FABP2 concentration was greater in Group A than in Group B suggesting that the intestinal mucosal dysfunction in the patients with chronic cholecystitis was more serious than that in the patients with acute appendicitis. Further research is required to explore the causes of the inconsistent changes in the D-lactic acid and FABP2 levels. There were no differences in the plasma LPS concentration between the two groups, but the postoperative levels of LPS in the two groups were lower than the preoperative levels. Cholecystolithiasis is related to abnormal intestinal mucosal barrier function leading to bacterial translocation, thus causing the formation of gallstones [23]. Inoculating guinea pigs with pathogenic bacteria involved in human cholecystitis resulted in increased permeability of the intestinal mucosal barrier and abnormal expression of structural proteins [24]. For patients with obstructive jaundice, preoperative probiotics can improve intestinal mucosal permeability after biliary drainage [25]. The intestinal mucosal dysfunction in patients with chronic cholecystitis may be related to the intestinal microbiome, which is associated with several clinical conditions [26,27]. Therefore, gallbladder diseases and changes in intestinal mucosal permeability can each reciprocally cause the other condition, but the specific mechanism remains unclear. in view of the damage to intestinal mucosa caused by chronic biliary diseases, the protection of intestinal function during the perioperative period should not be underestimated. Drugs that can improve the microcirculation of intestinal mucosa, such as dexmedetomidine, can be used during surgery [28].

Sdc-1 is the most important heparin sulfate proteoglycan on the cell surface and is mainly expressed on the lateral basal surface of epithelial cells. It plays an important role in maintaining cell morphology, promoting tissue repair, regulating immune function, and establishing cell-cell adhesion. Scd-1 is found associated with tight junctions (TJs) and participates in the maintenance and regulation of the intestinal mucosal barrier [29]. Supplementation with exogenous Sdc-1 enhances the expression of ZO-1 and occludin and synergizes with TJs to enhance barrier function and inhibit bacterial translocation. Sdc-1 is also an early target molecule for many pathogenic bacteria. The loss of Sdc-1 as a result of genetic mutation reduces the susceptibility of mice to several bacterial infections [30]. Sdc1 is essential for maintaining a normal epithelial barrier. Its exogenous exfoliation is regulated by inflammation, which induces an increase in the serum level of soluble Scd-1 (SSDC-1), suggesting an increase in intestinal mucosal permeability. Therefore, SSDC-1 might be a new marker of intestinal mucosal injury [31,32]. In this study, the postoperative Sdc-1 level was lower than the preoperative level, probably due to the effects of the anesthetics. The concentration of Sdc-1 in the patients with chronic cholecystitis was higher than that in the patients with appendicitis, indicating that the degree of damage to the intestinal mucosal barrier function of the former was higher than that of the latter, which was consistent with the results for FABP2. The postoperative inconsistency may be due to the different half-lives of the two indicators or the different inhibitory effects of the anesthetics.

The limitations of the study are that the intestinal permeability indicators at 24 hours, 48 hours, or longer post-operation were not observed, so they cannot reflect the degree of intestinal mucosal injury in the patients after surgery. When recruiting subjects, only septic shock patients were excluded without considering whether the appendix was perforated. In addition, we detected the LPS concentration but not the level of bacterial DNA. Our original hypothesis was that acute and chronic inflammation has different effects on intestinal mucosal function, and acute inflammation causes more serious intestinal mucosal damage. The results are beyond the original assumption. Although chronic cholecystitis has only chronic inflammation, the degree of gastric mucosal damage is more serious than that of acute appendicitis due to its special pathophysiological characteristics. Such a result is a fascinating part of this study. We will explore chronic cholecystitis versus acute cholecystitis or acute appendicitis versus chronic appendicitis on the intestinal mucosal barrier in further research.

## Conclusions

In summary, we concluded that the inflammation in patients with appendicitis undergoing laparoscopic appendectomy was stronger than that in the patients with cholecystitis or cholecystectomy, but the effect on intestinal mucosal permeability was contrary to the inflammation observations. Further research is needed to determine the specific mechanism of the effect of inflammation on intestinal mucosal permeability. The results of this study give clinicians some hints that for acute appendicitis, it is important to reduce inflammatory mediators to protect intestinal function. In view of the damage to intestinal mucosa caused by chronic biliary diseases, the protection of intestinal function during the perioperative period should not be underestimated.
